# The Association of Metabolic Syndrome with Diabetic Retinopathy: The Korean National Health and Nutrition Examination Survey 2008–2012

**DOI:** 10.1371/journal.pone.0157006

**Published:** 2016-06-08

**Authors:** Tai Kyong Kim, Jae Yon Won, Jeong Ah Shin, Yong-Moon Park, Hyeon Woo Yim, Young-Hoon Park

**Affiliations:** 1 Department of Ophthalmology and Visual Science, College of Medicine, The Catholic University of Korea, Seoul, Republic of Korea; 2 Department of Preventive Medicine, College of Medicine, The Catholic University of Korea, Seoul, Republic of Korea; 3 Department of Epidemiology and Biostatistics, Arnold School of Public Health, University of South Carolina, Columbia, South Carolina, United States of America; College of Medicine, National Taiwan University, TAIWAN

## Abstract

**Aims:**

To explore gender differences and associations between metabolic syndrome (MetS) and its components, and diabetic retinopathy (DR) in Korean adults aged 40 years and older with diabetes.

**Methods:**

We analyzed data from the Korean National Health and Nutrition Examination Surveys (2008–2012). In total, 2,576 type 2 diabetic participants, aged 40 and older, were evaluated. Seven standard retinal fundus photographs were obtained after pupil dilation in both eyes. DR was graded using the modified Airlie House classification system. Vision-threatening diabetic retinopathy (VTDR) included proliferative diabetic retinopathy and clinically significant macular edema. MetS was defined according to the Joint Interim Statement, proposed in 2009, by the International Diabetes Federation and the American Heart Association/National Heart, Lung, and Blood Institute. Multivariate logistic regression analysis was used to assess the relationship between MetS and its individual components with DR and VTDR.

**Results:**

After controlling for confounders, MetS was not associated with DR in men or women. Moreover, the risk for DR or VTDR did not increase with increasing MetS components. However, high waist circumference was significantly inversely associated with VTDR (adjusted odds ratio = 0.36; 95% confidence interval = 0.14–0.93) only in men.

**Conclusions:**

MetS was not associated with DR or VTDR in a Korean diabetic population. However, among MetS components, it seems that abdominal obesity was inversely associated with VTDR in Korean diabetic men.

## Introduction

The term metabolic syndrome (MetS) was introduced in 1988 by Reaven, who suggested that insulin resistance and compensatory hyperinsulinemia are responsible for the clustering of cardiovascular risk factors, such as glucose intolerance, hypertension, abdominal obesity, and dyslipidemia [1]. The presence of MetS predicts the risk of cardiovascular disease (CVD) in type 2 diabetes subjects as well as in non-diabetics [2, 3]. In addition to resulting in macrovascular complications, such as CVD, correlations between MetS and microvascular complications, including diabetic retinopathy (DR), have been shown in American and European subjects [4, 5].

However, in East Asian populations, such as Korean and Japanese, the situation is somewhat different from the Caucasian population. The prevalence of obesity differs markedly between Caucasians and East Asians in diabetics [6, 7], and the impact of obesity on CVD risk is also markedly different between Caucasians and East Asians [8]. Moreover, the predictive power of MetS is apparently lacking, even in CVD in Japanese diabetics [9]. Thus, it is doubtful as to whether the overall concept of MetS, based mostly on data from European and American patients, is clinically useful in the evaluation of the risk of DR in Eastern Asian diabetics.

There are few data on the relationship between MetS and DR in East Asian countries, and they are controversial. Terauchi et al. [10] found that neither the presence of MetS, as defined by the International Diabetes Federation (IDF) guidelines, nor an increased waist circumference (WC) increased the risk of DR in Japanese patients with type 2 diabetes mellitus (DM). In contrast, Shimajiri et al. [11] suggested that MetS was associated with DR in Japanese type 2 diabetic patients. Moreover, the risk of DR increased with the number of MetS components, rather than MetS *per se*, in a Chinese study [12]. However, none of these studies were population-based studies with large numbers of subjects.

Thus, in the present population-based study, we first assessed the association of MetS and its individual components with DR. Then, we assessed vision-threatening diabetic retinopathy (VTDR) in a representative Korean population.

## Materials and Methods

### Study Population

The present study was based on data acquired in the Korean National Health and Nutrition Survey (KNHNS), 2008–2012. The KNHNS, conducted annually by the Division of Chronic Disease Surveillance, Korean Center for Disease Control and Prevention, uses a rolling sampling design involving a complex, stratified, multistage, probability-cluster survey of a representative sample of the noninstitutionalized civilian population in Korea [13]. The survey consisted of a health interview, nutritional survey, and health examination survey. The survey collected data via household interviews and by direct standardized physical examinations conducted in a specially equipped mobile examination center. The sample design and size were estimated by KNHNS, so that the results could be generalized to the entire Korean population.

Annually, 4000 households in 200 enumerated districts are selected by a panel to represent the civilian, noninstitutionalized South Korean population using a systematic stratified, multistage-clustered sampling method, based on National Census Data. All of the members of each selected household were asked to participate in the survey, and the participation rates between 2008 and 2012 ranged from 77.8% to 82.8%.

All of the participants provided written informed consent. This study design followed the tenets of the Declaration of Helsinki for biomedical research and was approved by the Institutional Review Board of the Catholic University of Korea, Seoul, Korea.

Those who were diagnosed by a self-reported history of a physician diagnosis or were receiving drug treatment for DM, including insulin and oral hypoglycemic agents, and those who had a fasting plasma glucose level > 126 mg/dL were defined as diabetics. In the present analysis, we limited the study population to type 2 diabetic patients aged 40 years or older. Participants were considered to have type 1 DM if they were aged < 30 years when diagnosed with DM and were receiving insulin therapy [14]. Otherwise, DM was considered type 2 DM.

### Measurements

All of the participants were asked about their demographic and socioeconomic characteristics, including residential area, education, income, and occupation. Respondents who were exposed to sunlight for more than 5 h per day were categorized as the sun exposure group. Respondents were categorized into two groups: ever smokers (current smokers and ex-smokers) and non-smokers. High-risk drinking was defined as the consumption of at least five alcoholic drinks for men, or four or more alcoholic drinks for women, in a row, at least once in the past 2 weeks [15]. The subjects who engaged in moderate or vigorous exercise on a regular basis were designated as those who exercised regularly. For vigorous exercise, such as jogging, hiking, soccer, basketball, squash, etc., a regular basis was defined as more than three times per week for over 20 min each time. For moderate exercise, such as volleyball, badminton, table tennis, tennis doubles, etc., a regular basis was defined more than five times per week for over 30 min each time.

There was no item in the KNHNS that showed daily magnesium intake. However, the daily magnesium intake from foods was estimated using a magnesium content database reported by a previous study [16]. The subjects of the 2008~2012 KNHANES consumed 2,429 types of food based on 24-h dietary recall data. Regarding food intake, 59.3% of the items had information on magnesium content in the database. Foods not included in the magnesium content database were calculated by replacing them with similar foods in the database.

WC was measured to the nearest 0.1 cm in a horizontal plane at the level of the midpoint between the iliac crest and the costal margin at the end of a normal expiration. Body mass index (BMI) was calculated by dividing body weight by height squared (kg/m^2^) after height and weight were measured using standardized techniques and equipment. High BMI was defined as a BMI > 25 kg/m^2^. Blood pressure (BP) was measured three times on the right arm while the individual was in a seated position after at least 5 min of rest using a mercury sphygmomanometer (Baumanometer; Baum, Copiague, NY). The final BP value was obtained by averaging the values of the second and third BP measurements. Blood samples were collected in the morning after fasting. If fasting time was less than 8 h, the fasting glucose of that patient was considered a missing value, and if it was less than 12 h, triglycerides were considered a missing value. Serum levels of glucose, total cholesterol (TC), high-density lipoprotein (HDL) cholesterol, and triglycerides were measured enzymatically using a Hitachi automatic analyzer 7600 (Tokyo, Japan). Glycated hemoglobin (HbA_1c_) was measured by the SLS hemoglobin (no cyanide) method using a XE-2100D (Sysmex, Japan).

The duration of DM was calculated as the difference between the year of diagnosis and the examination year of KNHNS in known diabetic patients. Those who had a fasting plasma glucose level > 126 mg/dL without a previous diagnosis of DM were classified as subjects with new DM and were given a DM duration of zero years.

### Assessment of DR

Non-mydriatic fundus photography (TRC-NW6S; Topcon, Tokyo, Japan) was performed in all of the KNHNS participants. Seven standard field photographs according to the Early Treatment for Diabetic Retinopathy Study (ETDRS) protocol were obtained from each eye after pharmacological pupil dilation [17]. DR was identified if any characteristic lesion, defined by the ETDRS severity scale, was present. A DR severity score was assigned to each eye according to the modified Airlie House classification system [17–19]. The level of retinopathy was graded based on the worse eye. Eyes were graded according to the following criteria: no DR (level 10–13), non-proliferative diabetic retinopathy (NPDR) (level 14–51), and proliferative diabetic retinopathy (PDR) (level > 60). Clinically significant macular edema was defined according to the ETDRS criteria [20]. VTDR was defined as the presence of PDR or clinically significant macular edema; severe NPDR was not included in our study.

A preliminary grading was done on-site by ophthalmologists trained by the National Epidemiologic Survey Committee of the Korean Ophthalmologic Society (KOS). The fundus photos were re-examined by 15 university hospital professors (retina specialists). The quality of the survey was verified by the Epidemiologic Survey Committee of the KOS.

### Definition of MetS

MetS was defined using the criteria proposed by the American Heart Association (AHA) and the National Heart, Lung, and Blood Institute (NHLBI), together with the IDF, in 2009 [21]. MetS was defined as (1) a waist circumference ≥ 90 cm in men and ≥ 80 cm in women, according to the IDF criteria for Asian countries; (2) fasting glucose ≥ 100 mg/dL or being on medication for elevated glucose; (3) fasting triglycerides ≥ 150 mg/dL or cholesterol-lowering medication use; (4) HDL-cholesterol < 40 mg/dL in men and < 50 mg/dL in women or cholesterol-lowering medication use; and (5) systolic BP ≥ 130 mmHg and/or diastolic BP ≥ 85 mmHg or being on antihypertensive drug treatment for patients with a history of hypertension. A MetS diagnosis requires at least three of the five components to be present.

### Statistical Analyses

All of the data are presented as means ± SE for continuous variables or proportions (SE) for categorical variables. Statistical analyses were conducted using the SAS statistics software for Windows, version 9.2 (SAS Institute, Cary, NC, USA); the stratification and clustering of the design were included in the analyses of this study to ensure appropriate estimates and standard errors according to specific guidelines for analyses and reporting of sample weights that were provided by the KNHNS[13]. To minimize the effects of variation among survey years, all of the analyses performed in this study were adjusted for survey year. In addition, we conducted stratified analyses to assess effect modification by gender in the association of MetS and its components with DR and VTDR.

Simple logistic regression analyses were performed to estimate the magnitude of association of MetS, DR, and VTDR with the parameters of the participants. Multiple logistic regression analyses were performed to estimate the magnitude of the association of DR and VTDR with MetS and its components, and two statistical models based on the characteristics of the variables were used. One model included age and survey year. Subsequently, socioeconomic and lifestyle-related characteristics including income, education, residential area, smoking status, drinking alcohol, exercise, occupation (farmer or fisher), and sun exposure were included, based on the results from the univariate analysis. The Cochran–Armitage Trend Test was used to test the effects of clustering of MetS components on DR and VTDR. A P value < 0.05 was considered statistically significant.

## Results

In total, numbers of type 2 diabetic subjects were 2576 (weighted: 2,633,811): 1303 (weighted: 1,450,384) men and 1273 (weighted: 1,183,427) women. Of these, 2099 (82.7%) participants had non-mydriatic fundus photographs and 2046 (81.0%) participants had 7 gradable standard photographs. The prevalence of MetS in this population was 79.5% (95% confidence interval (CI) = 77.4–81.4%; 71.1% for men and 89.3% for women). The prevalences of individual components of MetS were 55.8% for abdominal obesity, 58.2% for elevated triglycerides, 64.4% for low HDL-cholesterol, and 69.4% for high BP. Table 1 shows the characteristics of the participants by MetS status.

**Table 1:** Characteristics according to the presence or absence of metabolic syndrome in diabetic patients by sex.

	Overall percentage	Total		P	Men		P	Women		P
		absence	presence		absence	presence		absence	presence	
Unweighted number		491	1985		371	850		120	1135	
Weighted number		517262	2005217		392802	964484		124460	1040733	
Sex (%)				**< .001**						
Male	53.8(1.2)	75.9(2.3)	48.1(1.3)							
Female	46.2(1.2)	24.1(2.3)	51.9(1.3)							
Age (years)				0.061			0.083			**0.001**
40–49	17.7(1.2)	22.4(2.7)	16.5(1.2)		20.0(3.1)	19.9(1.9)		30.1(5.5)	13.3(1.5)	
50–59	30.7(1.2)	31.4(2.9)	30.6(1.3)		32.2(3.4)	39.0(2.1)		29.0(5.3)	22.7(1.5)	
60–69	27.8(1.0)	25.0(2.1)	28.5(1.1)		27.3(2.6)	26.8(1.7)		17.6(3.3)	30.0(1.6)	
≥70	23.8(1.0)	21.2(2.0)	24.5(1.1)		20.5(2.2)	14.3(1.2)		23.3(4.6)	33.9(1.7)	
Residence (urban)	74.3(2.0)	71.1(3.1)	75.1(2.0)	0.111	71.5(3.4)	75.9(2.4)	0.173	69.8(5.7)	74.4(2.2)	0.375
Education (>6 years)	57.4(1.3)	68.3(2.5)	54.7(1.4)	**< .001**	74.3(2.7)	76.9(1.7)	0.409	49.3(5.7)	34.1(1.9)	**0.008**
Income (lowest quartile)	31.4(1.2)	28.6(2.5)	32.0(1.3)	0.220	29.2(3.1)	24.9(1.8)	0.219	27.0(4.8)	38.7(1.7)	**0.033**
Occupation (farmer/fisherman)	10.4(1.1)	12.8(2.0)	9.8(1.1)	0.091	13.5(2.4)	11.0(1.5)	0.311	10.6(3.9)	8.7(1.2)	0.595
Sun exposure (>5 hours/day)	23.0(1.3)	29.2(2.9)	21.5(1.3)	**0.004**	33.6(3.3)	26.5(1.9)	**0.042**	15.5(3.9)	16.8(1.6)	0.754
Heavy smoker (yes)	50.1(1.3)	66.9(2.8)	45.8(1.4)	**< .001**	85.5(2.3)	85.4(1.5)	0.992	9.1(3.3)	8.9(1.1)	0.965
Heavy drinker (yes)	11.7(0.9)	16.2(2.4)	10.5(1.0)	**0.016**	20.8(3.0)	20.6(1.8)	0.951	1.7(1.3)	1.2(0.6)	0.712
Regular exercise (yes)	20.4(1.0)	20.7(2.1)	20.4(1.1)	0.908	21.0(2.6)	24.0(1.8)	0.342	19.6(4.3)	17.0(1.4)	0.525
DM duration (≥5 years)	43.7(1.2)	45.5(2.9)	43.2(1.3)	0.453	46.8(3.4)	38.1(2.0)	**0.021**	41.4(5.7)	48.0(1.8)	0.283
DM control (HbA_1c_ <6.5%)	28.4(1.3)	33.2(2.8)	27.1(1.4)	**0.043**	34.4(3.3)	28.1(2.1)	0.096	29.3(5.2)	26.3(1.8)	0.584

DM, diabetes mellitus. Data are presented as the mean±SE, or % (SE). Significant P-values are in bold font.

The prevalence of DR in participants aged 40 years or older was 17.7% (95% CI = 15.719.8%; 17.6% for men and 17.7% for women). Table 2 shows the characteristics of the participants according to the presence or absence of DR. The prevalence of VTDR in participants aged 40 years or older was 3.2% (95% CI = 2.4–4.1%; 2.9% for men and 3.5% for women). Table 3 shows characteristics of the participants according to the presence or absence of VTDR.

**Table 2:** Characteristics according to the presence or absence of diabetic retinopathy in diabetic patients by sex.

	Overall percentage	Total		P	Men		P	Women		P
		absence	presence		absence	presence		absence	presence	
Unweighted number		1716	383		853	189		863	194	
Weighted number		1794397	384715		972488	207520		821908	177194	
Sex (%)				0.943						
Male	54.2(1.3)	54.2(1.4)	53.9(3.2)							
Female	45.8(1.3)	45.8(1.4)	46.1(3.2)							
Age (years)				**0.002**			**< .001**			0.053
40–49	20.0(1.3)	21.7(1.5)	12.1(2.3)		24.4(2.1)	11.4(3.2)		18.6(1.9)	13.0(3.3)	
50–59	33.1(1.3)	33.4(1.4)	31.4(3.1)		39.2(2.1)	40.0(4.6)		26.6(1.8)	21.3(4.1)	
60–69	27.2(1.0)	25.8(1.2)	33.5(2.8)		24.3(1.5)	33.9(3.9)		27.7(1.7)	33.0(3.8)	
≥70	19.7(0.9)	19.0(1.0)	23.0(2.4)		12.1(1.0)	14.7(2.4)		27.2(1.8)	32.7(4.3)	
Residence (urban)	74.8(2.1)	74.9(2.2)	74.1(3.3)	0.804	76.2(2.4)	73.8(4.6)	0.594	73.4(2.6)	74.6(4.0)	0.787
Education (>6 years)	59.6(1.4)	60.2(1.5)	56.9(3.1)	0.324	77.6(1.6)	75.5(4.3)	0.632	39.6(2.2)	35.2(4.4)	0.375
Income (lowest quartile)	28.3(1.2)	28.1(1.3)	29.4(2.8)	0.680	23.3(1.8)	20.9(3.3)	0.546	33.8(1.9)	39.5(4.4)	0.230
Occupation (farmer/fisherman)	10.7(1.2)	11.0(1.3)	9.2(2.5)	0.509	12.2(1.6)	9.1(3.6)	0.471	9.7(1.5)	9.3(2.6)	0.898
Sun exposure (>5 hours/day)	22.5(1.4)	23.2(1.6)	19.3(2.4)	0.178	28.1(2.0)	24.6(3.8)	0.404	17.3(1.9)	13.2(2.8)	0.248
Heavy smoker (yes)	49.2(1.3)	49.3(1.5)	49.1(3.3)	0.969	84.9(1.5)	83.3(3.3)	0.655	7.2(1.1)	9.3(2.7)	0.439
Heavy drinker (yes)	12.0(0.9)	12.0(1.0)	11.9(2.2)	0.970	20.8(1.7)	21.7(3.8)	0.832	1.7(0.7)	0.6(0.6)	0.316
Mg intake (lowest quartile)	25.1(1.3)	25.0(1.5)	25.4(2.9)	0.887	22.0(1.9)	23.1(4.3)	0.809	28.2(2.2)	28.0(4.0)	0.970
Regular exercise (yes)	21.8(1.2)	22.9(1.3)	16.4(2.3)	**0.019**	26.4(1.9)	15.0(3.2)	**0.008**	18.8(1.8)	17.9(3.4)	0.811
DM duration (≥5 years)	43.0(1.3)	36.8(1.4)	71.7(3.0)	**< .001**	34.3(2.0)	66.5(4.5)	**< .001**	39.8(2.1)	77.8(3.5)	**< .001**
DM control (HbA_1c_ <6.5%)	27.6(1.3)	31.1(1.5)	11.2(2.2)	**< .001**	33.1(2.2)	11.6(3.3)	**< .001**	28.7(2.0)	10.8(2.6)	**< .001**

Data are presented as the mean±SE, or % (SE). DM, diabetes mellitus; Mg, magnesium. Significant P-values are in bold font.

**Table 3:** Characteristics according to the presence or absence of vision-threatening diabetic retinopathy in diabetic patients by sex.

	Overall percentage	Total		P	Men		P	Women		P
		absence	presence		absence	presence		absence	presence	
Unweighted number		1966	80		979	35		987	45	
Weighted number		2065861	67581		1118000	33615		947860	33966	
Sex (%)				0.533						
Male	54.0(1.3)	54.1(1.4)	49.7(6.9)							
Female	46.0(1.3)	45.9(1.4)	50.3(6.9)							
Age (years)				0.289			0.160			0.547
40–49	20.3(1.3)	20.5(1.4)	11.7(4.3)		23.0(1.9)	9.1(6.1)		17.6(1.8)	14.3(5.9)	
50–59	33.3(1.3)	33.2(1.3)	37.6(7.2)		39.2(1.9)	48.0(10.8)		26.0(1.7)	27.3(7.9)	
60–69	27.0(1.1)	27.0(1.1)	24.6(5.2)		25.6(1.4)	25.8(7.8)		28.8(1.6)	23.6(6.6)	
≥70	19.5(0.9)	19.3(1.0)	26.0(5.8)		12.2(0.9)	17.2(6.5)		27.6(1.7)	34.7(8.5)	
Residence (urban)	74.7(2.1)	74.8(2.1)	70.6(6.2)	0.452	75.5(2.4)	79.4(8.4)	0.659	74.0(2.5)	61.9(8.6)	0.113
Education (>6 years)	59.6(1.4)	60.1(1.4)	44.1(6.9)	**0.020**	77.7(1.5)	61.5(11.4)	0.095	39.3(2.1)	27.4(7.8)	0.176
Income (lowest quartile)	28.3(1.2)	28.1(1.3)	35.8(6.4)	0.214	23.0(1.6)	22.8(8.6)	0.988	34.1(1.8)	48.6(8.5)	0.083
Occupation (farmer/fisherman)	10.9(1.2)	10.9(1.2)	11.8(4.7)	0.835	11.9(1.5)	13.8(7.3)	0.771	9.8(1.4)	9.8(6.0)	0.993
Sun exposure (>5 hours/day)	22.7(1.4)	22.8(1.4)	18.4(5.0)	0.402	28.2(1.9)	21.1(8.3)	0.442	16.5(1.7)	15.8(5.6)	0.895
Heavy smoker (yes)	49.4(1.4)	49.5(1.4)	48.2(7.0)	0.860	85.1(1.4)	87.6(5.0)	0.643	7.6(1.0)	9.2(4.7)	0.725
Heavy drinker (yes)	12.0(1.0)	12.3(1.0)	4.2(2.1)	0.022	21.5(1.6)	8.5(4.4)	**0.044**	1.5(0.6)	0(0)	0.633
Mg intake (lowest quartile)	25.1(1.3)	24.9(1.4)	30.8(6.1)	0.329	22.1(1.8)	27.0(8.4)	0.554	28.0(2.0)	34.9(8.6)	0.403
Regular exercise (yes)	21.8(1.2)	22.0(1.2)	16.7(5.1)	0.362	24.8(1.7)	19.5(8.4)	0.571	18.6(1.7)	13.9(5.8)	0.466
DM duration (≥5 years)	42.3(1.3)	40.9(1.3)	85.0(5.3)	**< .001**	37.6(2.0)	80.3(8.6)	**< .001**	44.8(2.0)	89.7(6.1)	**< .001**
DM control (HbA_1c_ <6.5%)	27.9(1.4)	28.3(1.4)	16.7(6.4)	0.139	30.0(2.0)	24.0(11.5)	0.633	26.2(1.8)	10.0(4.8)	**0.024**

DM, diabetes mellitus; Mg, magnesium. Data are presented as the mean±SE, or % (SE). Significant P-values are in bold font.

After controlling for confounding factors, MetS was not significantly associated with DR or VTDR in either gender (Table 4). One criterion for MetS, increased fasting plasma glucose ≥ 100 mg/dL or pre-existing diabetes, was present in all of the participants in our study due to our study design. As a result, the OR of this component could not be calculated, and this component was excluded from the table. In the component analysis for MetS, only high WC showed an inverse association with VTDR in men; no other significant association was found in either gender. Table 5 shows that the prevalence of DR and VTDR did not change significantly with an increase in the number of MetS components in either gender.

**Table 4:** Association of metabolic syndrome and its components with diabetic retinopathy in diabetic patients by sex.

Variables	Diabetic retinopathy				Vision-threatening diabetic retinopathy			
	Model 1^a^		Model 2^b^		Model 1^a^		Model 2^b^	
	OR (95% CI)	P	OR (95% CI)	P	OR (95% CI)	P	OR(95% CI)	P
Men								
MetS (presence vs. absence)	0.67(0.43,1.06)	0.087	0.69(0.41,1.16)	0.163	0.54(0.23,1.30)	0.171	0.44(0.16,1.20)	0.108
MetS component								
High WC	0.69(0.46,1.03)	0.072	0.78(0.48,1.26)	0.304	0.41(0.15,1.14)	0.086	**0.36(0.14,0.93)**	**0.036**
Low HDL	1.07(0.71,1.62)	0.743	0.88(0.55,1.43)	0.612	0.86(0.37,2.01)	0.733	0.51(0.20,1.34)	0.172
High triglycerides	0.99(0.63,1,55)	0.967	0.94(0.56,1.60)	0.823	0.70(0.29,1.71)	0.433	0.76(0.28,2.12)	0.603
High blood pressure	0.82(0.53,1.26)	0.361	0.99(0.61,1.60)	0.972	0.64(0.28,1.50)	0.304	0.75(0.27,2.05)	0.572
Other variables								
High BMI	0.62(0.41,0.97)	0.034	0.74(0.44,1,23)	0.246	0.40(0.15.1.11)	0.078	0.34(0.10,1.10)	0.071
Systolic BP(continuous variable)	1.01(1.00,1.02)	0.212	1.01(1.00,1.03)	**0.045**	0.99(0.96,1.03)	0.704	1.00(0.97,1.03)	0.873
Diastolic BP(continuous variable)	0.98(0.96,1.00)	0.082	0.99(0.97,1.02)	0.581	0.95(0.92,0.98)	0.001	0.97(0.93,1.01)	0.122
Women								
MetS (presence vs. absence)	0.65(0.35,1.20)	0.166	0.56 (0.29,1.12)	0.101	1.18(0.35,3.98)	0.786	1.88(0.34,10.43)	0.881
MetS component								
High WC	0.80(0.51,1.25)	0.325	0.67(0.40,1.12)	0.124	0.61(0.29,1.28)	0.189	0.66(0.29,1.50)	0.314
Low HDL	1.12(0.67,1.87)	0.674	0.90 (0.52,1.57)	0.706	1.48(0.60,3.70)	0.396	1.61(0.51,5.13)	0.420
High triglycerides	1.49(0.98,2.26)	0.061	1.44(0.90,2.30)	0.130	1.33(0.62,2.85)	0.463	1.36(0.57,3.28)	0.490
High blood pressure	0.68(0.43,1.08)	0.103	0.83 (0.51,1.37)	0.475	0.53(0.25,1.15)	0.107	0.65(0.26,1.60)	0.347
Other variables								
High BMI	0.59(0.40,0.87)	0.008	0.57(0.36,0.91)	**0.018**	0.58(0.30,1.14)	0.115	0.61(0.29,1.27)	0.189
Systolic BP(continuous variable)	1.01(1.00,1.02)	0.176	1.01(1.00,1.03)	**0.036**	1.01(0.99,1.03)	0.604	1.01(0.99,1.03)	0.208
Diastolic BP(continuous variable)	0.98(0.95,1.00)	0.031	0.99(0.97,1.02)	0.423	0.97(0.94,1.01)	0.105	1.00(0.96,1.04)	0.936

Data are presented as the odds ratio (95% confidence interval).

^a^Model 1 was adjusted by age.

^b^Model 2 was adjusted by socioeconomic and lifestyle-related characteristics including income, education, residential area, smoking status, drinking alcohol, exercise, occupation (farmer or fisherman), sun exposure, diabetes mellitus duration, and serum HbA1c level.

MetS, metabolic syndrome (vision-threatening diabetic retinopathy, proliferative diabetic retinopathy and clinically significant macular edema); WC, waist circumference; HDL, high density lipoprotein cholesterol; BP, blood pressure; BMI, body mass index; OR, odds ratio; CI, confidence interval. Significant P-values are in bold font.

**Table 5:** Contribution of cumulative metabolic syndrome components in diabetic retinopathy and vision-threatening diabetic retinopathy by sex.

Variables	Diabetic retinopathy				Vision-threatening diabetic retinopathy			
	Model 1^a^		Model 2^b^		Model 1^a^		Model 2^b^	
	OR (95% CI)	P	OR (95% CI)	P	OR (95% CI)	P	OR (95% CI)	P
Men								
Number of MetS components								
0~1	1		1		1		1	
2	0.53(0.29,0.96)		0.51(0.26,0.99)		0.56(0.16,1.92)		0.58(0.13,2.60)	
3	0.83(0.45,1,53)		0.84(0.43,1.68)		1.04(0.33,3.26)		0.78(0.18,3.35)	
4	0.93(0.49,1.76)		0.88(0.40,1.94)		0.09(0.01,0.78)		0.08(0.01,0.79)	
P for trend	0.631		0.660		0.356		0.189	
Women								
Number of MetS components								
0~1	1		1		1		1	
2	0.41(0.20,0.83)		0.35(0.16,0.80)		1.23(0.32,4.76)		1.80(0.33,9.91)	
3	0.82(0.41,1.64)		0.71(0.33,1.52)		1.44(0.38,5.46)		1.95(0.38,9.89)	
4	0.71(0.35,1.45)		0.61(0.27,1.34)		0.62(0.15,2.57)		0.67(0.12,3.69)	
P for trend	0.861		0.617		0.477		0.563	

Data are presented as the odds ratio (95% confidence interval).

^a^Model 1 was adjusted by age.

^b^Model 2 was adjusted by socioeconomic and lifestyle-related characteristics including income, education, residential area, smoking status, drinking alcohol, exercise, occupation (farmer or fisherman), sun exposure, diabetes mellitus duration, and serum HbA1c level.

MetS, metabolic syndrome (vision-threatening diabetic retinopathy, proliferative diabetic retinopathy and clinically significant macular edema); OR, odds ratio; CI, confidence interval.

## Discussion

MetS, defined according to the Joint Interim Statement proposed in 2009, was not associated with the presence of DR or VTDR complications in Korean patients with type 2 diabetes. Even a clustering of MetS components did not lead to an increase in the prevalence of DR or VTDR. In contrast, the Metascreen Writing Committee reported that the presence of MetS, as defined by the National Cholesterol Education Program (NCEP) and IDF guidelines, was a strong, independent predictor of DR in type 2 diabetics [4]. Furthermore, other studies of Caucasians have consistently shown the same relationship between MetS and DR [5, 22].

Data on the relationship between MetS and DR in East Asian populations are less consistent. One clinical cross-sectional study based on 130 Japanese patients reported the lack of an association between MetS and DR, similar to our results [10]. In contrast, some studies in East Asia have shown conflicting results. Pang et al. [12] showed that MetS, as defined by the Chinese Diabetes Society, was highly associated with DR in Chinese subjects, rather than the IDF or ATP III definitions, and that the risk for DR increased with the aggregation of three or more MetS components; this was a community-based study of the Chinese population with a sample size of 3,240 participants. One clinical cross-sectional study, based on 637 Japanese patients by Shimajiri et al. [11], suggested a positive association between MetS and DR in type 2 Japanese diabetic patients.

However, these two conflicting studies had some problems in their statistical analyses in controlling for compounding factors, such as DM duration and HbA_1c_. DM duration and HbA_1c_ are well-known risk factors for DR according to previous reports, and this was also shown in our study [23–26]. Thus, it is essential to control for the effects of these factors when studying other risk factors for DR; we controlled for these factors and socioeconomic factors by including them in the multiple logistic regression model. Although Pang et al. [12] used multinomial logistic regression in their analysis, important confounding factors, such as DM duration, HbA_1c_, and socioeconomic factors, were not included in their model, and the robustness of their results is questionable. Shimajiri et al. [11] subdivided their subjects by DM duration, but they did not control for other factors, such as HbA_1c_ and socioeconomic factors, using a multiple logistic regression model.

The differences between our study and reports based on Caucasian populations may be explained, at least in part, by possible ethnicity-related differences in the role of the insulin resistance, represented by MetS in development of microvascular complications of DM. Both insulin deficiency and insulin resistance are involved in the pathogenesis of type 2 DM. Moreover, the relative balance of the factor may differ widely by ethnicity.

Because insulin resistance is considered a key player in the pathophysiology of the MetS [1, 27], the strong relationship with DR and MetS in Caucasians reflects the importance of insulin resistance in this ethnicity. In contrast, many previous studies support a relatively more important role of an insulin secretory defect, compared with insulin resistance, in type 2 diabetic subjects in East Asians, including Koreans and Japanese. In contrast to diabetic patients among Caucasians, most of whom have a higher degree of obesity than non-diabetics, most type 2 diabetic patients in Korea are not obese, and many of them lose significant weight during the course of developing diabetes [7]. In addition, the peak plasma insulin level was much lower than that of Caucasians, whereas insulin sensitivity in first-degree relatives of Korean type 2 diabetic subjects was not lower than in control subjects [28]. Moreover, other studies have reported that an insulin secretory defect, rather than insulin resistance, may play an important role in the development of type 2 diabetes in Koreans [29, 30]. Taken together, these reports from Korea perhaps suggest that the insulin secretory capacity of Korean people, who are smaller than Western people, cannot compensate for the insulin resistance resulting from recent changes in their life style.

Similar non-obese characteristics and the relative importance of an insulin secretory defect in type 2 DM were shown in Japanese studies [6, 31]. Because Koreans are genetically close to the Japanese and have experienced recent rapid economic changes, it is possible that similar mechanisms may be operating in the pathogenesis of glucose intolerance in Japanese subjects.

Type 1 DM and type 2 DM patients of East Asian origin share the same feature, with an insulin secretory defect that is more dominant, although insulin resistance is also involved in the development of the disease. Due to this, MetS appears to have little predictive value in distinguishing type 1 diabetic patients who are most likely to develop DR, even in Caucasians. Kilpatrick et al. [32] showed that the IDF definition of MetS appeared to have little clinical utility in distinguishing type 1 diabetic patients who are most likely to develop microvascular disease, including DR, in the Diabetes Control and Complications Trial (DCCT). The Metascreen Writing Committee reported that the presence of MetS, as defined by the NCEP and IDF guidelines, did not show a statistically significant association with DR in type 1 diabetic patients, whereas, in contrast, MetS was a strong, independent predictor of DR in type 2 diabetic patients in this study [4]. These examples of type 1 DM also showed the restricted role of MetS on DR in DM patient groups that showed a relative dominance of an insulin secretory defect over insulin resistance.

In the analysis of MetS components, no component showed a positive association with DR or VTDR in our study. In particular, a high WC showed an *inverse* relationship with VTDR in men. In women, there was a significant *inverse* relationship between high BMI and DR. In men, a high BMI had a negative relationship with VTDR, but it was not significant by a narrow margin (P = 0.071).

These findings contrast with many previous reports in which obesity was a risk factor for DR [33–37]. However, in the Wisconsin Epidemiologic Study of Diabetic Retinopathy (WESDR), when controlling other risk factors, older-onset persons in the WESDR who were underweight at baseline were three times more likely to develop retinopathy as those of normal weight, consistent with our results [38]. Other studies have also reported associations of lower BMI with DR [25, 39].

High WC patients had short durations of diabetes in our study (P = 0.045), which may partly explain the inverse relationship between high WC and VTDR in our study. Significant weight loss was also observed in another study during the course of the development of diabetes in a Korean population, also consistent with our study [7]. However, this does not fully explain the seemingly protective effect of WC on VTDR in males, because after controlling DM duration in model 2, the protective effect was still seen in the multiple logistic regression analysis.

Several studies have suggested the so-called obesity paradox, which is an apparently protective effect of obesity against adverse clinical outcomes in patients with obstructive coronary artery disease [40–42]. One of these studies explained this by an anti-inflammatory effect of obesity [40]. Adipose tissue is now recognized as a major endocrine organ and obesity is associated with high serum levels of low-density lipoproteins that scavenge unbound circulating lipopolysaccharides, and thus have anti-inflammatory effects [43, 44]. Because an inflammatory component is also considered a characteristic of DR, especially macular edema [45], this may also support the seemingly protective effect of high WC on VTDR.

Hypertension is the one of the most consistent risk factors of DR in many studies, although the details differ in studies. In the prospective Wisconsin study, systolic BP predicted progression of retinopathy in patients under 30 years of age at onset [46]. The United Kingdom Prospective Diabetes Study [47] reported that the occurrence of retinopathy was associated with higher systolic BP, consistent with other studies in subjects of various ethnicities [24–26, 48].

Although hypertension as a component of MetS did not show an association with DR or VTDR in our study, DR was associated with higher systolic BP in both genders after controlling for other significant factors. Thus, there may be a problem with the criteria for MetS rather than hypertension *per se* not being a risk factor for DR in Koreans. If we revise the criteria for MetS, focusing on systolic BP and discarding diastolic BP, this criterion may have clinical significance as a risk factor for DR in Koreans.

Associations of DR with hyperlipidemia are less consistent than with hypertension. Serum lipid profiles were associated with DR or VTDR in our study, consistent with some previous studies [24, 48, 49]. However, data from the ETDRS showed that higher levels of serum lipids (LDL cholesterol, very LDL cholesterol, and triglycerides) were associated with an increased risk of hard exudates in the macula and vision loss in persons with diabetes [50]. In the WESDR, higher serum total cholesterol was associated with a higher prevalence of retinal hard exudates in both younger- and the older-onset groups taking insulin but not in those with type 2 diabetes using oral hypoglycemic agents [51]. Further prospective studies with larger sample sizes in East Asians are necessary to address this issue.

Our study had some limitations. One was that a causal relationship could not be assessed because of the cross-sectional nature of study. We attributed the lack of influence of MetS on DR to the balance between insulin resistance and insulin secretory defects, but markers that can be used to evaluate insulin secretory ability, such as the insulinogenic index or C-peptide levels, were not measured in our study. Further prospective studies to measure these surrogate markers of insulin secretory ability in East Asians may confirm our hypothesis.

Although our study used criteria to exclude type 1 diabetes, it is possible that type 1 diabetic patients could have been included. However, considering the extremely low incidence of type 1 diabetes in Korea (1.36 cases per year per 100,000 individuals), the probability is minimal [52].

Zimmet introduced the term ‘‘latent autoimmune diabetes of adults” (LADA) to describe a subgroup of adult phenotypic Type 2 diabetic patients positive for glutamic acid decarboxylase (GADA) [53]. Several studies reported that LADA patients, compared with Type 2 diabetic patients, have fewer markers of MetS [54, 55]. Because GADA was not investigated in the KNHNS, the percentage of LADA patients was not available in our study. Based on findings in the UKPDS, 10% of adults with diabetes have LADA [56]. If the percentage of LADA in Koreans was higher than Western societies, the lack of association between DMR and MetS in our study could be attributed to a high percentage of LADA, which would affect the conclusion of the study. However, it has been reported that the prevalence of GADA positive patients with Type 2 diabetes in Korea was 1.7–5.3% [57]. This prevalence tends to be lower than that of Western countries.

Despite these limitations, this study used a nationally representative sample of adults in Korea with a high response rate, a crucial strength of our study. Moreover, we controlled other risk factors statistically, which can affect the results versus other previous studies dealing with the same issues in East Asians with fewer or less complete controls. Moreover, to our knowledge, this is the first reported large population-based study to examine the association between DR and VTDR with MetS and its components in East Asians.

In conclusion, the presence of MetS, defined according to the Joint Interim Statement proposed in 2009, was not associated with the presence of DR or VTDR in a representative Korean population with type 2 diabetes. VTDR was inversely associated with a high WC in men. As a consequence, this study showed that classifying a patient with Type 2 diabetes with MetS may be of limited or no use in predicting DR or VTDR.
